# Neuropeptides, Altruism, and Adverse Childhood Experiences: Investigating Biological and Behavioral Correlations in Medical Students

**DOI:** 10.3390/brainsci15101128

**Published:** 2025-10-21

**Authors:** Jennifer Khong, Lauren Bennett, Johanna Felix Rivera, Nathan Andrews, Veronica Vuong, Demi Zapata, Phillip Khong, Rebecca Ryznar

**Affiliations:** 1 Department of Internal Medicine, Banner Desert Medical Center, Mesa, AZ 85201, USA; jennifer.khong@bannerhealth.com; 2 Department of Emergency Medicine, Texas Tech University Health Sciences Center, Lubbock, TX 79430, USA; lauren.bennett@ttuhsc.edu; 3 College of Osteopathic Medicine, Rocky Vista University, Parker, CO 80112, USA; nathan.andrews@co.rvu.edu; 4 Department of Pediatrics, Wake Forest University, Winston-Salem, NC 27109, USA; veronica.vuong@co.rvu.edu; 5 Department of Otolaryngology, Geisinger Health System, Danville, PA 17822, USA; dnzapatagerkin@geisinger.edu; 6 Department of Interventional Radiology, HCA HealthONE Sky Ridge, Lone Tree, CO 80124, USA; phillip.khong@co.rvu.edu; 7 Department of Biomedical Sciences, Rocky Vista University, Parker, CO 80112, USA; rryznar@rvu.edu

**Keywords:** neuropeptides, CLS-H, ACE score, altruism, stress, substance P, hypothalamic–pituitary–adrenal axis

## Abstract

**Background/Objectives**: This pilot study aimed to investigate the relationship between salivary neuropeptides levels, adverse childhood experiences (ACEs), and altruism in a sample of medical students. Additionally, the study examined potential sex differences in these relationships. **Methods**: Sixty medical students (36.6% men, 63.3% women) provided saliva samples to measure oxytocin, α-MSH, β-endorphin, neurotensin, and substance P using a custom 5-plex human peptide assay. Participants completed the ACE Survey and Compassionate Love Scale for Humanity (CLS-H) Altruism Survey. Descriptive statistics characterized demographics and survey data, with out-of-range values adjusted to the standard curve maximum. Data normality was assessed with the Jarque–Bera test; due to non-normality, values were log-transformed. Differences between male and female salivary, ACE score, and CLS-H altruism score were tested using *t*-tests and Mann–Whitney U-tests, while correlations were evaluated with Pearson and Spearman coefficients. **Results**: The five neuropeptides, while highly correlated with each other, did not exhibit significant relationships with altruism, as measured by the CLS-H Altruism Survey. Finally, female participants demonstrated greater altruistic tendencies compared with male participants with marginal significance. **Conclusions**: While there were no significant relationships between the fives neuropeptides, ACEs, or altruism; women demonstrated higher levels of altruism compared with men. The data reported in this pilot study did not strongly support the conclusion that neuropeptides influence social behavior and trauma response. Furthermore, future studies with larger, more diverse samples and multiple time point measurements of neuropeptides could be beneficial to better understand the relationships between neuropeptides and any potential implications for mental health interventions.

## 1. Introduction

The exploration of neuropeptides and their profound impact on psychological processes has garnered significant attention within the field of psychology. Neuropeptide functions are determined by a myriad of factors including their primary sequences, peptide lengths, proteolytic processing, and post-translational modifications [1]. Oxytocin, substance P, alpha-melanocyte stimulating hormone (α-MSH), beta-endorphin (β-endorphin), and neurotensin (NT) are neuropeptides that function as neurotransmitters and hormones in cell–cell communication within neuroendocrine systems [2]. Understanding the functions of these molecules enhances our comprehension of the biological underpinnings of behavior, emotion regulation, and social interactions [3]. This interdisciplinary area of study, bridging neuroscience and psychology, offers invaluable insights into the mechanisms underlying various psychological phenomena and informs the development of targeted therapeutic interventions. We illustrate some proposed neuropeptide mechanisms in Supplementary Figure S1.

The term altruism has a diffuse usage and is thought to encompass behaviors that are counterproductive to self-preservation but secure the preservation of someone or something else [4]. Some examples of this can include donations of assets or resources, risking one’s life to secure another, payment of taxes, and caring for individuals unrelated to oneself [4]. The five neuropeptides investigated in this study function to regulate social behavior, influence altruism, mitigate stress, and reinforce reward pathways. In addition to its well-known role in uterine contractions and milk letdown during childbirth and breastfeeding, oxytocin aids in fostering cooperation, feeling empathy, and facilitating emotional attachment while also mediating anxiety and cortisol release during social stress [5,6,7]. Empathy can be defined as the propensity to share and understand the feelings of others upon interactions cognitively and emotionally [8]. In contrast to empathy, compassion is a concept with a wider range that can be felt at larger scales in response to suffering and involves thoughts, emotions, and behaviors directed at others [9]. Similar to the positive functions of oxytocin, β-endorphin is integral to the brain’s internal reward system, enhancing dopamine and serotonin release, potentially inducing a sense of euphoria during altruistic acts [4,10]. GABA is a primary inhibitor of dopamine release in the brain. In reward pathways, β-endorphin binds to μ-opioid receptors and inhibits GABA. This leads to an excess accumulation of dopamine from the lack of inhibition and thus the feelings of euphoria and well-being [10]. It also mediates the release of corticotropin-releasing hormone (CRH) in response to illness and augments immune function [11]. Finally, β-endorphin as well as substance P, α-MSH, and NT have associations with pain regulation and trauma response. β-Endorphin’s analgesic effect is achieved by increasing the pain threshold through the inhibition of nociceptors [10]. In addition to mitigating inflammation and nociception, elevated substance P is associated with antisocial traits, depression, impulsivity, and has been implicated in chronic inflammatory conditions such as asthma and migraines [12,13,14,15]. Substance P release is triggered by inflammation or injury, and it has the highest affinity to NK1 receptors in the brain. Upon binding, a metabolic cascade is initiated that ultimately leads to an increase in intracellular calcium that enhances various physiological pathways. Pain and stress thresholds are lowered in neurons, thus eliciting enhanced responses to stimuli [15]. Substance P is also known to be localized in regions of the brain that are associated with depression, meaning that the excitatory activity of the neurotransmitter in those regions can contribute to depressive symptoms [15]. α-MSH, involved in neuroimmunomodulation and behavioral arousal, shows varying levels in individuals with trauma, influencing adaptive social behaviors including altruism [16,17]. NT impacts stress-related disorders by acting on the hypothalamic–pituitary–adrenal (HPA) axis and subsequent cortisol release [18]. The HPA axis plays a crucial role in regulating responses to stress [19]. NT also acts upon dopamine and glutamate, influencing reward-related behaviors such as substance use and has modulatory effects that can lead to maladaptive behaviors under chronic stress [19]. In chronically stressed rats, the effects of neurotensin were seen to be sensitized within a month. In those same rats, blocking neurotensin receptors pharmacologically showed a reduction in anxious behavior [19]. This suggests that the neuropeptide could be working to stimulate the HPA axis during stress conditions, inhibiting dopamine pathways and increasing anxiety [19,20].

Given the role of neuropeptides in regulating stress responses and social behaviors, it becomes crucial to understand how early environmental factors, such as adverse childhood experiences (ACEs), might influence these biological pathways and consequently impact long-term health outcomes. Adverse childhood experiences can be described as any abuse, domestic violence, or household dysfunction that takes place during childhood [21]. Childhood abuse can be categorized as physical, sexual, psychological, or emotional [21]. The Adverse Childhood Experiences (ACE) survey is currently endorsed by the Centers for Disease Control and Prevention (CDC) as a valuable tool for predicting health risks, as exposure to ACEs is strongly linked to poor physical and mental health outcomes in adulthood [21]. For example, having higher ACE scores is associated with worse cardiovascular health as well as substance abuse, depression, anxiety, and suicidal tendencies [21,22]. This well-established relationship illuminates how negative early life experiences can have enduring effects on the brain, increasing the risk of both physical and neuropsychiatric diseases [23,24]. Further corroborating this association, epigenetic changes to genes regulating stress response and the immune system have been observed in trauma survivors, potentially explaining the lasting and transgenerational impacts of trauma [24]. While some studies demonstrate that trauma can impair the development of empathy and altruism, other research suggests that suffering can lead to an increase in these traits, a phenomenon known as “altruism born of suffering” [25,26].

ACEs have profound implications on both physical and psychological health. Investigating the potential effects of ACEs on neuropeptides like oxytocin, substance P, α-MSH, β-endorphin, and NT may underscore the importance of early life experiences shaping the human neuroendocrine system and subsequent social behaviors like altruism. This pilot study investigated the connection between salivary levels of these neuropeptides, recorded ACE scores, and levels of altruism. Based on prior literature linking adverse childhood experiences to dysregulated stress responses, we hypothesized that higher ACE scores would be associated with elevated substance P levels and reduced oxytocin levels. Furthermore, we anticipated that higher oxytocin and β-endorphin levels would correlate with increased altruism, while elevated substance P would be linked to lower altruism.

## 2. Materials and Methods

### 2.1. Participants

The study enrolled 96 participants, recruited from Rocky Vista University College of Osteopathic Medicine in Colorado in October 2022. Of the 96 participants initially enrolled, 36 were excluded from the final analysis due to data integrity issues. Specifically, 13 participants had incomplete neuropeptide assay results, 5 had missing survey responses, and 14 could not be included due to misidentified ID codes that prevented linkage across datasets. These exclusions were attributable to methodological errors rather than participant-related factors, and there is no evidence to suggest that excluded participants differed demographically or behaviorally from those included in the final analysis. The study was non-selective regarding biological sex, gender identity, age, or racial and ethnic identity. Most participants were between the ages of 22 and 35, with two outliers at 48 and 61 years. The inclusion criteria required participants to be at least 18 years old, willing and able to provide consent, and able to perform saliva collection for the study. Individuals who were pregnant or breastfeeding were excluded due to oxytocin fluctuations during these times. Racial and ethnic information was not collected from the participants.

### 2.2. Procedure

The study took place on the Rocky Vista University, Colorado campus in Parker, Colorado. Upon enrollment in the study, each participant was assigned a unique four-digit identifier by a research team member not involved in data analysis. Each participant was given verbal and written information about the study and signed a consent form on the day of participation. Female subjects were provided with a pregnancy test and were required to show research personnel their negative test results prior to participating. All subjects were asked to refrain from eating or drinking anything besides water two hours prior to their participation. Saliva collection occurred during a single time window in the afternoon, scheduled according to participant availability and convenience. While this approach limited our ability to account for diurnal fluctuations in neuropeptide levels, it ensured consistency in timing across participants within the cohort.

Participants first provided saliva samples by allowing approximately 7.5 mL of saliva to passively flow into a 15 mL centrifuge tube. Saliva samples were mixed with 1 uL/mL Sigma protease inhibitor cocktail, then stored at −20 °C for stabilization. Participants then filled out surveys in the following order on a secure, password-protected laptop: the Compassionate Love Scale for Humanity (CLS-H), the Perceived Stress Scale (PSS), and the Adverse Childhood Experiences (ACE) questionnaire. The PSS served as a control measure, as individuals with unusually high levels of stress may subsequently have altered levels of oxytocin and β-endorphin. The PSS results were not used for data analysis purposes. While the research team calculated the scores of the CLS-H and PSS for each participant, the study subjects calculated and reported their ACE scores through an anonymous online survey using their four-digit identifier. Responses to the CLS-H and PSS surveys were also recorded through an anonymous online survey associated with each subject’s four-digit identifier. The total participation time for each subject was approximately 20 min, and subjects were compensated with a 20 USD gift card for their time.

### 2.3. Neuropeptide Saliva Level

Saliva samples (1 mL) were collected using SafeCollect Saliva Collection Kits (Greiner Bio-One, Kremsmünster, Austria). To preserve peptide stability, Sigma-Aldrich Protease Inhibitor Cocktail (Cat. #P8340; diluted at 1 μL/mL saliva) was added immediately after collection. Participants fasted for at least 2 h and rinsed their mouths prior to collection to reduce contamination. Following collection, samples were stored at −20 °C for 2–6 weeks prior to shipment. All samples were packed on dry ice and shipped overnight in insulated containers to Eve Technologies (Calgary, AB, Canada) in November 2022. Upon arrival, samples were logged and stored at −80 °C until batch analysis, which was completed within 30 days. Neuropeptide concentrations were quantified using a custom 5-plex human peptide assay (Eve Technologies, Calgary, AB, Canada) targeting oxytocin, α-MSH, β-endorphin, neurotensin, and substance P. This assay demonstrates a sensitivity range of 11–479 pg/mL, with high specificity and negligible cross-reactivity among analytes. Detailed assay specifications are provided in Supplementary Table S5. 

### 2.4. Questionnaires

The Compassionate Love Scale for Humanity (CLS-H) consists of 21 questions designed to assess an individual’s capacity to respond to the needs of others, serving as an indirect measure of altruism. Participants respond to each item on a seven-point Likert scale, ranging from 1 (not at all true of me) to 7 (very true of me). An average score is calculated across all questions, with higher scores indicating greater compassion and altruism. This survey was selected due to its strong reliability and validity [9].

To analyze the various aspects of altruism, the questions were grouped into four main categories: (1) compassion and empathy (survey items 1, 3, 4, 9, 12, 15, 17, 18), which reflect the cognitive and emotional ability to understand the feelings of others; (2) altruistic motivation (survey items 2, 6, 10, 13, 21), which refers to the internal drive to engage in helping behaviors; (3) altruistic behavior (survey items 5, 7, 8, 11, 19, 20), representing intentional actions that benefit others, even at personal cost; and (4) non-judgmental acceptance (survey items 14, 16), defined as the capacity to understand others without prejudice. Additionally, the questions were categorized based on the target of altruism: toward strangers, globally, and toward oneself. In the preliminary study that informed this research, no predefined categories were established for the purpose of each question. Using rational inference and keyword analysis, the research team collaboratively grouped the questions into their appropriate subgroups to assist with analysis and explore how different aspects of altruism may influence other variables being studied.

The Perceived Stress Scale is a 10-question survey designed to assess an individual’s perceived stress level. Developed in 1983, this survey poses questions that assess one’s perceived control, coping abilities, and overall mood over the past month. Each question is scored on a five-point Likert scale, ranging from 0 (never) to 4 (very often). Scores of zero to 13 indicate low perceived stress, 14–26 indicate moderate perceived stress, and 27–40 indicate high perceived stress. This survey was chosen due to its demonstrated internal consistency and reliability when administered across diverse populations [27,28]. Therefore, no data analysis was conducted using the PSS test, and no significant results were drawn from it. Finally, the Adverse Childhood Experiences (ACE) questionnaire consists of 10 yes or no questions assessing one’s exposure to various negative, early life events, or circumstances that can make childhood exceptionally difficult. Developed in 1985 by Dr. Vincent Felitti, this survey is now recognized by the CDC as an important tool in assessing the long-term physical health risks and is utilized in many healthcare settings in the United States for this purpose. The CLS-H, ACE, and PSS can be found in Appendix A.1, Appendix A.2, and Appendix A.3, respectively.

### 2.5. Data Analysis

Analysis was conducted in Rstudio version 2022.12.0+353. Descriptive statistics were generated to characterize demographics, survey data, and salivary data displayed in Supplementary Tables S1 and S2. All reported concentrations with values more than the standard curve were reduced to the highest observed concentration within the standard curve. Data were evaluated for Gaussian distribution by the Jarque–Bera test statistic displayed in Supplementary Table S3. Due to the non-normal distribution of cytokines, values were scaled using logarithm base 10. With a highly specific sample composed of limited size, both parametric and non-parametric tests were used to best uncover all potential relationships. Both a two-sided independent *t*-test and two-sided Mann–Whitney U-test were used to evaluate male/female differences and the Pearson correlation coefficient and Spearman rank correlation were used to evaluate for relationships.

## 3. Results

Sixty participants composed the study cohort. Of the participants, 38 (63%) were female and 22 (36%) were male, with a mean age of 27.7 ± 7.5 years. Supplementary Table S4 contains the participant ID, biological sex, CLS-H survey score, ACE score, perceived stress score, and neuropeptide levels.

### 3.1. Survey Data Analysis

**Figure 1:**
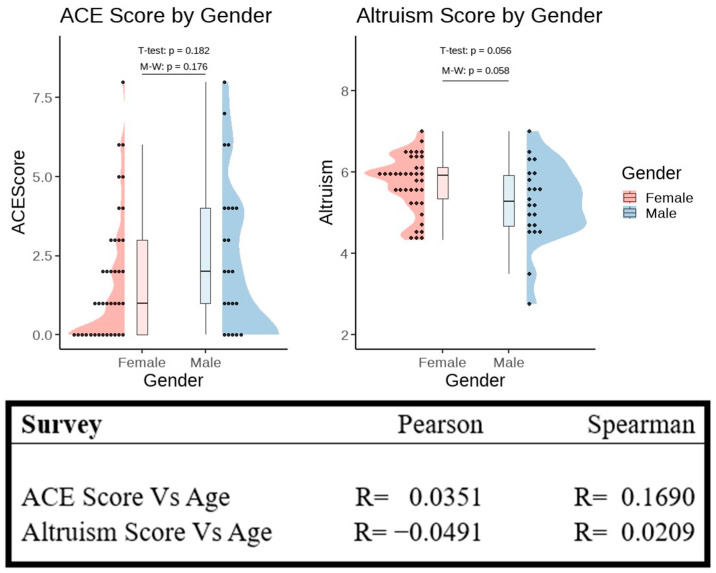
Raincloud plots of ACE and altruism scores by gender. Dots show individual data; half-violins display the distribution, and boxplots represent the summary of statistics. Brackets indicate *t*-tests and Mann–Whitney U-tests; *p*-values are shown with significance denoted by asterisks. Pearson and Spearman correlations between the survey scores and age are listed in the table.

Participants completed the CLS-H to measure degrees of altruism as well as the ACE survey to assess childhood trauma. Correlation coefficients (with their *p*-values) with respect to age are displayed in Figure 1. Results from the *t*-test and Mann–Whitney U-statistic with respect to biological sex are also displayed in Figure 1. Our data showed no correlation between age and survey scores in our population group. Female participants, however, trended toward significantly higher scores on the CLS-H compared with male participants (*t*-test: *p* = 0.056; Mann–Whitney: *p* = 0.058).

### 3.2. Neuropeptide Evaluation and Interrelationships

**Figure 2:**
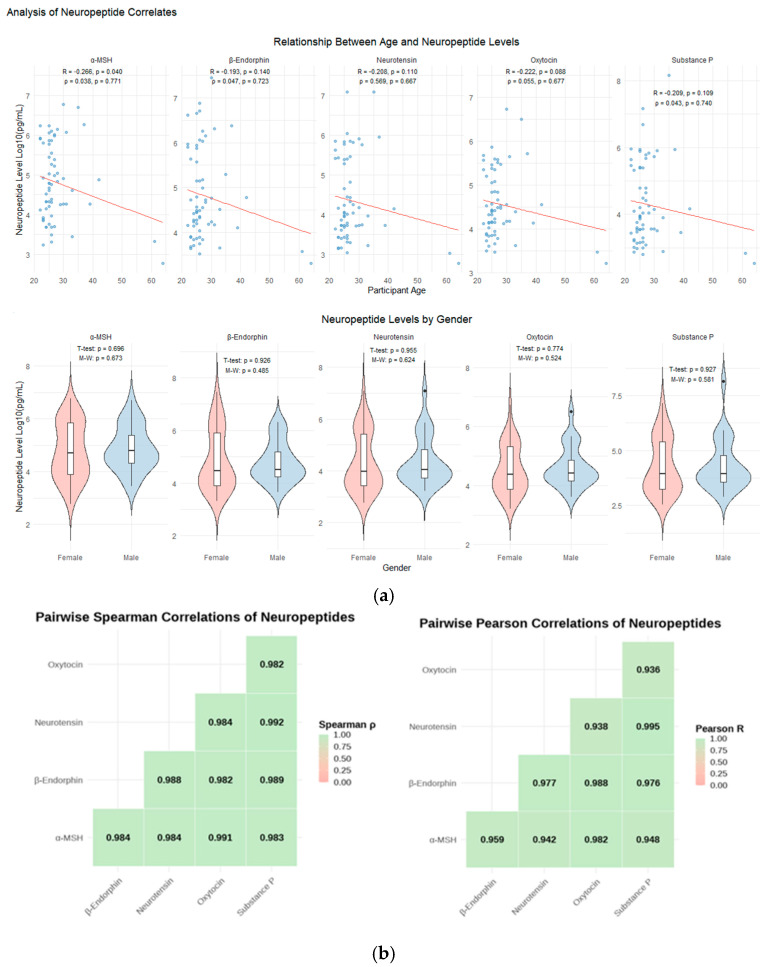
(**a**) Pearson and Spearman correlation coefficients between the five neuropeptides and participant age are displayed in the first row. The second row displays neuropeptide differences from genders using *t*-tests and Mann–Whitney U-tests as (**b**) Heatmaps of pairwise correlations between salivary neuropeptide levels. Pearson (R) and Spearman (ρ) correlation coefficients are shown separately. Values are displayed within each tile, with coefficients greater than 0.9 shown in bold.

Relationships between salivary neuropeptide concentrations and collected demographics are displayed in Figure 2a. Similar to before, these were calculated for both parametric and non-parametric tests, as described in the Methods section. α-MSH was negatively correlated with the participants’ age (*t*-test: R = −0.266; *p* = 0.040), while the rest of the neuropeptides similarly trended toward a negative correlation with age.

To assess the interrelationship of our neuropeptides, both a Pearson and Spearman correlation coefficient matrix were generated, assessing each combination of neuropeptides. The results are shown in Figure 2b. Pearson correlation coefficients revealed coefficients greater than R = 0.93 for all neuropeptide combinations. Spearman correlation coefficients displayed coefficients greater than ρ = 0.98 for all combinations.

**Figure 3:**
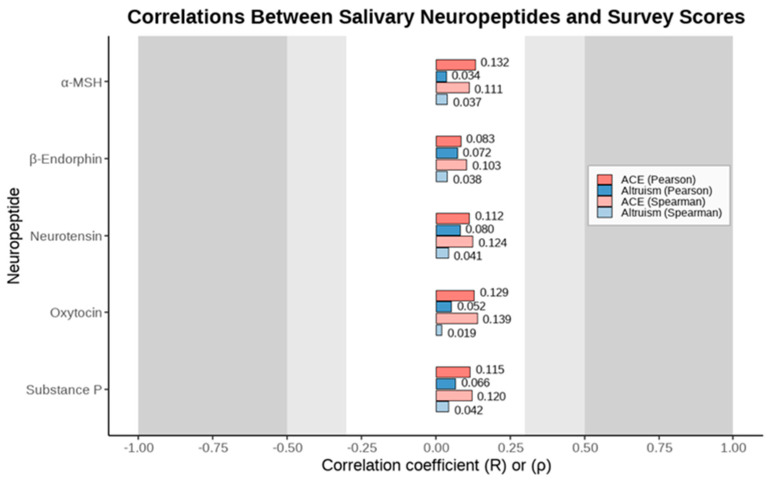
Correlations between salivary neuropeptide levels and survey scores. Pearson (darker bars) and Spearman (lighter bars) correlation coefficients are shown for both ACE and altruism surveys across five neuropeptides. Positive and negative correlations are displayed on a common scale (−1 to 1). Coefficients greater than |0.30| have been put in bold.

In order to evaluate a neuropeptide’s correlation with the participants’ survey results, additional Pearson and Spearman correlational coefficients were generated in Figure 3. None of the five neuropeptides displayed a strong correlation with respect to the participants’ survey results.

### 3.3. Identifying Motives in Survey Responses

Given the observed association between biological sex and altruistic responses, we conducted a post hoc exploratory subdivision of the CLS-H items. Survey questions were grouped into reconstructed categories (e.g., underlying motivation and stranger vs. worldview) based on shared wording and thematic content. This approach was intended to provide a more nuanced view of altruism by examining whether specific situational or motivational dimensions could account for the observed differences. As this subdivision was not based on prior validation studies, it should be considered exploratory and interpreted with caution.

**Figure 4:**
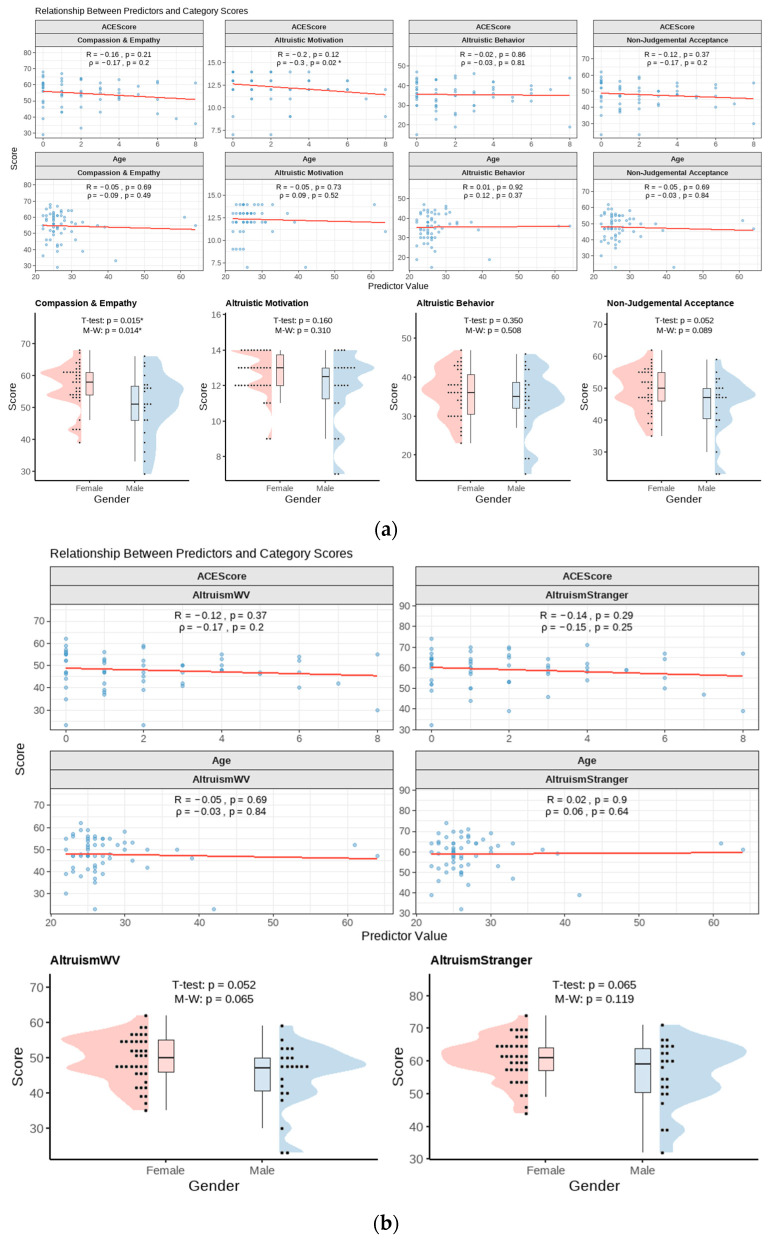
(**a**) Analysis after four sub-category creations from the altruism survey. Scatter plots (top, middle) show the linear correlation of ACE scores and age with each altruism survey sub-category score, displaying both the Pearson (R) and Spearman (ρ) correlation coefficients and their respective *p*-values. Raincloud plots (bottom) compare scores by gender, reporting results from both *t*-tests and Mann–Whitney U tests. Values with *p* < 0.05 are denoted with an asterisk (*). (**b**) Analysis after two sub-category creations from the altruism survey: Altruistic tendencies to the world and altruistic tendencies to strangers. Scatter plots (top, middle) show the linear correlation of ACE scores and age with each altruism survey sub-category score, displaying both the Pearson (R) and Spearman (ρ) correlation coefficients and their respective *p*-values. Raincloud plots (bottom) compare scores by gender, reporting results from both the *t*-tests and Mann–Whitney U tests. The dots are the participants, the line is a trendline. Values with *p* < 0.05 are denoted with an asterisk (*).

To thoroughly evaluate a participant’s life experience, the CLS-H, which served to assess one’s degree of altruism, was split into four categories based on each question’s underlying motivations. Pearson and Spearman correlation coefficients between age and ACE score are displayed in Figure 4a. The *p*-values from the *t*-test and Mann–Whitney statistic evaluating male and female scores are also listed for each category.

In both parametric and non-parametric tests, females exhibited significantly higher scores on questions relating to compassion and empathy compared with males (*p* = 0.015 and *p* = 0.014, respectively). Similarly, in questions relating to non-judgmental acceptance, females trended toward higher scores compared with males in both parametric and non-parametric tests (*p* = 0.052 and *p* = 0.089, respectively). When correlating with the ACE scores, questions relating to altruistic motivation were negatively correlated when using the Spearman correlation (ρ = −0.3, *p* = 0.02). A similar result was seen when using the Pearson correlation, however, not trending toward significance (R = −0.2, *p* = 0.12).

Questions from the CLS-H were also sub-categorized based on whether the question pertains to a stranger or humanity as a whole. Pearson and Spearman correlation coefficients between age and ACE score are displayed in Figure 4b. The *p*-values from both the parametric and non-parametric tests evaluating male and female scores are also listed for each category. Female participants trended toward higher scores relating to empathy toward strangers and toward the world compared with males while using parametric studies (*p* = 0.052 and *p* = 0.065, respectively). In non-parametric studies, a similar observation was seen with females trending toward higher scores in questions pertaining to an outlook on humanity (*p* = 0.065). No relationships of significance were found for this survey sub-categorization while using non-parametric studies.

## 4. Discussion

### 4.1. Neuropeptide and ACE Scores

In this study, we examined correlations between salivary levels of five neuropeptides—oxytocin, α-MSH, β-endorphin, neurotensin, and substance P—and the participants’ altruism levels and adverse childhood experiences (ACE). While we initially hypothesized that α-MSH, β-endorphin, neurotensin, and substance P would positively correlate with the ACE scores and negatively with altruism, our results showed no significant associations between these neuropeptides and the measured behaviors. Oxytocin, specifically, did not display any correlation with ACE scores (R = 0.129) and no correlation with altruism (R = 0.052) under a normal distribution. This could potentially be due to confounding factors like the single-point saliva collection, which may not fully capture the average neuropeptide levels over time. Additionally, differences in neuropeptide response between sexes were considered, as past studies have reported mixed findings like higher plasma oxytocin and β-endorphin in females [29,30]. A 2023 review looking at oxytocin measurements in saliva found that although saliva levels of oxytocin did not reflect the plasma levels, they were highly correlated with the CSF levels [6]. The review also highlighted that saliva sampling does reflect the concentration changes in stressful situations [6]. Substance P showed no correlation with ACE scores under normal distribution assumptions (R = 0.115) and non-normal distribution (ρ = 0.120). Future research should explore larger, more diverse samples and employ repeated neuropeptide measurements to clarify these relationships and reduce limitations in assessing the neuropeptide influence on social behavior and trauma response.

Oxytocin, α-MSH, β-endorphin, substance P, and neurotensin form a dynamic network that regulates stress, emotional resilience, and social behaviors, especially post-trauma. Oxytocin, α-MSH, and β-endorphin mitigate stress, promoting recovery and resilience, while substance P and neurotensin activate the HPA axis, intensifying stress responses [7,18]. Furthermore, alpha-MSH interacts with the oxytocin system in the hypothalamus, suggesting a mechanism by which peptides can influence complex behaviors [31]. Thus, the role these neuropeptides have on each other to regulate emotional response post stress exposure is something to consider in association with the ACE scores. Alternatively, childhood trauma has been known to alter levels of α-MSH, β-endorphin, ACTH, and cortisol [16]. Trauma during early childhood has also been known to result in altered emotional responses, risk-seeking behavior, and prevalence of psychiatric disease [16]. Adverse childhood experiences have a dose–responsive relationship with the probability of lifetime and recent depressive disorders [21]. This relationship suggests an associated increased risk of psychiatric disorders even decades after their occurrence [21]. The stria terminalis is a limbic pathway that connects the amygdala to the hypothalamus. Chronic stressors increase the volume, branching, and alterations of excitatory synaptic pathways in the Stria terminalis [19]. This is similar to the lowered thresholds seen in elevated substance P levels, thus contributing to increased depressive symptoms. Oxytocin’s effects on amygdala reactivity highlight its nuanced role in emotional regulation [32]. These findings underscore the intricate interplay among neuropeptides in shaping stress responses, emotional regulation, and resilience, offering a foundation for exploring their potential as biomarkers and therapeutic targets in trauma-related emotional and social dysfunctions.

### 4.2. Altruism and Biological Sex

This is example 1 of an equation: One notable incidental finding involves the relationship between biological sex and altruism. Female participants demonstrated a statistically significant increase in their scores on CLS-H altruism surveys compared with male participants (*p* = 0.056). Additionally, females scored significantly higher on questions specifically related to compassion and empathy, with an even more pronounced difference (*p* = 0.015). These findings correlate with current research indicating higher levels of empathy and altruism in women compared with men, observable from as early as primary school [8].

The exact reason behind this is difficult to pinpoint. Some studies suggest that this may stem from increased societal expectations for women to act more selflessly, while others propose that women may have an increased motivation to engage in altruistic acts [33]. Interestingly, not every component of altruism (such as altruistic motivation, altruistic behavior, and non-judgmental acceptance) displayed significant differences between the sexes within this study (Figure 4a). These findings, along with established studies, emphasize that biological sex’s role in altruism remains a complex and nuanced field of study. This multifaceted nature further underscores the need for additional investigation to fully elucidate the mechanisms underlying male and female differences in prosocial behaviors.

This study had several limitations that should be considered when interpreting the findings. First, the sample size was relatively small and homogeneous, consisting solely of medical students and faculty, which limits generalizability to broader populations. Second, saliva collection was performed using a single, non-standardized protocol without flow rate control, which may have introduced variability due to pH differences and could have affected the neuropeptide measurements. Moreover, it remains unclear whether salivary neuropeptide concentrations primarily reflect plasma ultrafiltration, local production in salivary glands, or a combination of both. These result limitations could be a result of the previously mentioned limitations in sampling population and collection protocols.

### 4.3. Neuropeptides in Saliva

In this pilot study, all saliva samples were collected at a single afternoon time point, scheduled based on participant availability. While this approach limited our ability to account for potential diurnal variation in neuropeptide levels, it did offer internal consistency by ensuring that all samples were obtained under similar contextual conditions. The absence of repeated or time-standardized sampling constrains the generalizability of our findings. Similar studies have reported comparable methodological challenges, highlighting the importance of collecting samples at multiple time points and using longitudinal designs to more accurately capture associations with psychiatric symptoms such as depression [34]. A 2022 study looking at salivary cytokines and hormones in acute stress response used the HD-42 Cytokine Plex Panel and the HD-6 Steroid/Thyroid Hormone Plex assay. These assays measured cytokines IL-6, IL-10, TNF-α, IFN-γ, and hormones cortisol, estradiol, progesterone, T3, T4, and testosterone [35]. In 2024, a study looking into salivary neuropeptide shifts in acute stress responses used a Luminex200 system to measure oxytocin, α-MSH, β-endorphin, neurotensin, and substance P and found a moderate positive correlation between all neuropeptides and resilience scores [18]. Taking a different approach, a 2019 study looking at neuropeptide roles in mental health and a 2020 study looking at childhood trauma severity and stress and satiety hormone levels both used serum levels to make their measurements, each with different collection and testing criteria. The 2019 results using fasting samples and ELISA found a significant association between substance P and the antisocial scale results for participants while the 2020 results using Immulite test kits found significant changes in ACTH and cortisol but not in β-endorphin and MSH [14,16]. Studies looking at the measurements of oxytocin, specifically in saliva, found discrepancies between saliva and blood but a high correlation between saliva and CSF [6]. Although these discrepancies in correlations were present, it was also found that saliva concentrations of neuropeptides do change in stressful situations and that not requiring a medical or lab setting avoids unnecessary stressors that interfere with measurements [6].

The correlation coefficients near 1.0 found between neuropeptides in our results could reflect true co-regulation or a lack of assay specificity. Because the Eve Technologies multiplex panel used in this study is internally validated for negligible cross-reactivity (<0.5%) among the five neuropeptides within the tested concentration range (11–479 pg/mL), the strong inter-peptide correlations we found likely reflect biological co-release within shared neuroendocrine pathways rather than antibody cross-reactivity. We acknowledge that this alternative explanation is a limitation in our study and future work should include single-analyte ELISAs and mass-spectrometry confirmation to verify analyte independence.

Saliva collection occurred during a single afternoon time window, scheduled based on participant availability and convenience. While this approach ensured consistency in timing across the cohort, it limited our ability to account for potential diurnal fluctuations in neuropeptide levels. For instance, one study in young adult women reported a significant increase in salivary oxytocin from awakening to early afternoon [36], while other noted that single baseline measurements of salivary oxytocin at rest may lack stability and may not reliably reflect the trait levels, underscoring the sensitivity of oxytocin to collection timing and contextual factors. Although there is less direct evidence regarding salivary α-MSH, β-endorphin, neurotensin, and substance P exhibiting morning peaks, neuropeptides often follow circadian rhythms similar to those of other peptide hormones, which typically reach peak concentrations at specific times after waking [37]. 

The absence of standardized collection timing or a multi-time point sampling design in the present study may have limited our ability to detect physiologically meaningful fluctuations in neuropeptide levels. These limitations could have contributed to the null findings observed and underscore the importance of incorporating repeated or time-controlled sampling strategies in future research to better account for the diurnal dynamics of salivary neuropeptides. To enhance the reliability and interpretability of neuropeptide measurements, future research should incorporate standardized collection procedures and repeated sampling across different time points.

Finally, approximately one-third of enrolled participants were excluded due to incomplete neuropeptide assays, missing survey data, or mismatched identifiers. These losses were attributable to methodological errors rather than participant-related factors, and there is no evidence to suggest systematic demographic or behavioral differences between excluded and retained participants. However, the reduction in sample size may still affect the robustness and generalizability of the findings.

Future research should recruit larger and more diverse samples, incorporate standardized and repeated sampling protocols, and account for environmental and stress-related variables to improve the accuracy and interpretability of salivary neuropeptide measurements.

## 5. Conclusions

This pilot study explored the relationship between salivary neuropeptides, adverse childhood experiences (ACEs), and altruism in medical students. While our initial hypotheses anticipated multiple associations, our findings revealed no significant correlation between neuropeptide, ACE, and altruism, but that there is a correlation present within neuropeptides themselves. Additionally, female participants demonstrated higher altruism scores compared with males, particularly in the domains of compassion and empathy.

These results suggest potential sex-related differences in altruistic tendencies and the difference of neuropeptides when compared with each other. Certain associations between ACEs and specific neuropeptides, such as oxytocin and substance P, may have reached statistical significance if not for the study’s limited samples of size. The reliance of single time point saliva collection and the homogeneity of the participant pool further underscore the need for a cautious interpretation of these findings. Future research with expanded cohorts and repeated neuropeptide sampling will be essential to clarify whether these observed trends are robust and clinically meaningful.

## Data Availability

The original contributions presented in the study are included in the article/Supplementary Materials; further inquiries can be directed to the corresponding author. The data are not publicly available due to their containing information that could compromise the privacy of the research participants.

## Supplementary Materials

The following supporting information can be downloaded at: https://www.mdpi.com/article/10.3390/brainsci15101128/s1, Figure S1: illustrates the proposed molecular mechanisms for each neuropeptide and corresponding psychological condition; Table S1: Descriptive Statistics data; Table S2: Demographics Data; Table S3: Jarque-Bara Statistic; Table S4: Participant Demographics and Scores; Table S5: ELISA Data Sensitivity and Specificity.

## Abbreviations

The following abbreviations are used in this manuscript:

ACEs	Adverse childhood experiences
CLS-H	Compassionate Love Scale for Humanity
α-MSH	Alpha-melanocyte stimulating hormone
NT	Neurotensin

## Appendix A

### Appendix A.1. The Compassionate Love Scale for Humanity

1. When I see people I do not know feeling sad, I feel a need to reach out to them.

not at all true of me  1 2 3 4 5 6 7 very true of me

2. I spend a lot of time concerned about the well-being of humankind.

not at all true of me  1 2 3 4 5 6 7 very true of me

3. When I hear about someone (a stranger) going through a difficult time, I feel a great deal of compassion for him or her.

not at all true of me  1 2 3 4 5 6 7 very true of me

4. It is easy for me to feel the pain (and joy) experienced by others, even though I do not know them.

not at all true of me  1 2 3 4 5 6 7 very true of me

5. If I encounter a stranger who needs help, I would do almost anything I could to help him or her.

not at all true of me  1 2 3 4 5 6 7 very true of me

6. I feel considerable compassionate love for people from everywhere.

not at all true of me  1 2 3 4 5 6 7 very true of me

7. I would rather suffer myself than see someone else (a stranger) suffer.

not at all true of me  1 2 3 4 5 6 7 very true of me

8. If given the opportunity, I am willing to sacrifice in order to let the people from other places who are less fortunate achieve their goals.

not at all true of me  1 2 3 4 5 6 7 very true of me

9. I tend to feel compassion for people even though I do not know them.

not at all true of me  1 2 3 4 5 6 7 very true of me

10. One of the activities that provides me with the most meaning to my life is helping others in the world who need help.

not at all true of me  1 2 3 4 5 6 7 very true of me

11. I would rather engage in actions that help others, even though they are strangers, than engage in actions that would help me.

not at all true of me  1 2 3 4 5 6 7 very true of me

12. I often have tender feelings toward people (strangers) when they seem to be in need.

not at all true of me  1 2 3 4 5 6 7 very true of me

13. I feel a selfless caring for most of mankind.

not at all true of me  1 2 3 4 5 6 7 very true of me

14. I accept others whom I do not know even when they do things I think are wrong.

not at all true of me  1 2 3 4 5 6 7 very true of me

15. If a person (a stranger) is troubled, I usually feel extreme tenderness and caring.

not at all true of me  1 2 3 4 5 6 7 very true of me

16. I try to understand rather than judge people who are strangers to me.

not at all true of me  1 2 3 4 5 6 7 very true of me

17. I try to put myself in a stranger’s shoes when he or she is in trouble.

not at all true of me  1 2 3 4 5 6 7 very true of me

18. I feel happy when I see that others (strangers) are happy.

not at all true of me  1 2 3 4 5 6 7 very true of me

19. Those whom I encounter through work and public life can assume that I will be there for them if they need me.

not at all true of me  1 2 3 4 5 6 7 very true of me

20. I want to spend time with people I do not know well so that I can help enrich their lives.

not at all true of me  1 2 3 4 5 6 7 very true of me

21. I very much wish to be kind and good to fellow human beings.

not at all true of me  1 2 3 4 5 6 7 very true of me

Scoring: An average score is calculated for all 21 items.

### Appendix A.2. The Adverse Childhood Experiences Questionnaire

While you were growing up, during your first 18 years of life:

1. Did a parent or other adult in the household often: swear at you, insult you, put you down, or humiliate you? Or act in a way that made you afraid that you might be physically hurt?

If Yes, enter 1 _____

2. Did a parent or other adult in the household often push, grab, slap, or throw something at you? Or ever hit you so hard that you had marks or were injured?

If Yes, enter 1 _____

3. Did an adult or person at least 5 years older than you ever touch or fondle you or have you touch their body in a sexual way? Or attempt or actually have oral, anal, or vaginal intercourse with you?

If Yes, enter 1 _____

4. Did you often feel that no one in your family loved you or thought you were important or special? Or your family did not look out for each other, feel close to each other, or support each other?

If Yes, enter 1 _____

5. Did you often feel that you did not have enough to eat, had to wear dirty clothes, and had no one to protect you? Or Your parents were too drunk or high to take care of you or take you to the doctor if you needed it?

If Yes, enter 1 _____

6. Were your parents ever separated or divorced?

If Yes, enter 1 _____

7. Were any of your parents or other adult caregivers often pushed, grabbed, slapped, or had something thrown at them? Or sometimes or often kicked, bitten, hit with a fist, or hit with something hard? Or ever repeatedly hit over at least a few minutes or threatened with a gun or knife?

If Yes, enter 1 _____

8. Did you live with anyone who was a problem drinker or alcoholic, or who used street drugs?

If Yes, enter 1 _____

9. Was a household member depressed or mentally ill, or did a household member attempt suicide?

If Yes, enter 1 _____

10. Did a household member go to prison?

If Yes, enter 1 _____

ACE score (Total “YES” answers): _______

### Appendix A.3. The Perceived Stress Scale

For each question, choose from the following alternatives:

0—Never; 1—Almost never; 2—Sometimes; 3—Fairly often; 4—Very often

________ l. In the last month, how often have you been upset because of something that happened unexpectedly?
________ 2. In the last month, how often have you felt that you were unable to control the important things in your life?
________ 3. In the last month, how often have you felt nervous and stressed?
________ 4. In the last month, how often have you felt confident about your ability to handle your personal problems?
________ 5. In the last month, how often have you felt that things were going your way?
________ 6. In the last month, how often have you found that you could not cope with all the things that you had to do?
________ 7. In the last month, how often have you been able to control irritations in your life?
________ 8. In the last month, how often have you felt that you were on top of things?
________ 9. In the last month, how often have you been angered because of things that happened that were outside of your control?
________ 10. In the last month, how often have you felt difficulties were piling up so high that you could not overcome them?

Figuring your PSS score. You can determine your PSS score by following these directions:

- First, reverse your scores for questions 4, 5, 7, and 8. On these 4 questions, change the scores like this: 0 = 4, 1 = 3, 2 = 2, 3 = 1, 4 = 0.
- Now add up your scores for each item to get a total. My total score is ___________.
- Individual scores on the PSS can range from 0 to 40 with higher scores indicating higher perceived stress.

► Scores ranging from 0 to 13 would be considered low stress.
► Scores ranging from 14 to 26 would be considered moderate stress.
► Scores ranging from 27 to 40 would be considered high perceived stress.
