# Supplementary material for: Neuropeptides, Altruism, and Adverse Childhood Experiences: Investigating Biological and Behavioral Correlations in Medical Students
Source: Brain Sci. 2025 Oct 21;15(10):1128. doi: 10.3390/brainsci15101128 (PMC12564630; doi:10.3390/brainsci15101128)
Supplement: Supplementary file 1 [file brainsci-15-01128-s001.zip › brainsci-3847864-supplementary.pdf]

Supplemental Figures

Supplemental Table S1– Descriptive Statistics data

Surveys

Variable	Mean	Std Dev	N	Median	Minimum	Maximum
Altruism Score	5.5482	0.8294	60	5.6190	2.7619	7
ACE Score	2.1500	2.2047	60	1.5	0	8

Neuropeptides

Variable	Mean	Std Dev	N	Median	Minimum	Maximum
α-MSH	410501	876646	60	51774.71	589.85	5938900
ß-Endorphin	963687	3751045	60	30667.97	2031.32	28550000
Neurotensin	151721	272385	60	9725.46	592.36	1108400
Oxytocin	194542	701786	60	25959.58	1634.43	5440700
Substance P	224803	658679	60	8301.11	338.36	4860900

Supplemental Table S1 shows descriptive statistics for the collective data for participant survey responses as well as for each neuropeptide that was measured from saliva samples.

Supplemental Table S2 – Demographics Data

	Number	%
Gender		
Male	22	36.6
Female	38	63.3
Age		
20-29	48	80.0
30-39	9	15.0
41-99	3	5.0

n=60

Supplemental Table S2 shows grouping percentages of the participants in terms of gender as well as age range.

Supplemental Table S3 – Jarque-Bara Statistic

Variable	Skewness	Kurtosis	JB Statistic
Altruism Score	-0.7993	1.001	8.897
ACE Score	1.0107	0.209	10.325

Variable	Skewness	Kurtosis	JB Statistic
α-MSH	4.497	25.95	1885.73
ß-Endorphin	6.839	49.97	6710.67
Neurotensin	1.897	2.605	52.96
Oxytocin	7.144	53.513	7669.34
Substance P	6.020	41.46	4659.7

Supplemental Table S3 shows the results of the Jarque-Bara statistical analysis of the collective participant data for survey scores as well as for each neuropeptide measured in saliva samples.

Supplemental Table S4 – Participant Demographics and Scores

ID	Age	Gender	CLH-S Score	Perceived Stress	ACE Score	OXY Saliva Level (pg/mL)	a-MSH (pg/mL)	B Endorphin (pg/mL)	Neurotensin (pg/mL)	Substance P (pg/mL)
1023	39	Female	5.2	13	5	13667.54	18273.63	12748.61	5304.66	2843.29
1507	27	Male	6.5	20	2	68371.86	242778.2	150885.89	66435.27	61064.52
1559	24	Female	6.5	18	1	6796.94	4862.35	6986.79	2519.78	1400.12
1560	26	Male	4.571429	14	6	71201.95	187017.1	143059.18	27985.98	30776.11
1571	27	Male	4.952381	18	0	329709.41	951288.7	1088000	699843.78	603891.51
1585	24	Female	5.190476	25	4	10692.99	14175.88	14085.94	4049.62	2924.46
1598	24	Female	6.761905	13	0	4076.27	4881.19	5047.51	1468.84	938.42
1600	25	Female	5.809524	24	0	26913.86	62279.82	36414.38	11336.21	9085.81
1618	23	Female	6	14	5	162466.92	641164.1	429147.08	270320.78	211759.17
1621	23	Female	5.571429	12	0	7717.62	8320.46	8055.37	2733.67	1773.02
1625	26	Famale	6.5	21	1	25952.47	57051.47	30429.34	10186.61	10947.58
1627	25	Male	5.333333	19	1	25966.68	61155.53	28246.35	10928.74	8492.18
1631	26	Male	2.761905	33	0	14204.45	20807.47	22598.87	5387.96	3596.65
1637	27	Male	6.285714	20	4	379749.91	1014000	1809500	290027.09	470920.84
1646	42	Male	3.5	8	2	35728.4	74329.97	59599.52	14254.25	14776.83
1648	27	Female	6.142857	31	8	30594.72	95981.5	36127.6	21163.05	26239.18
1655	37	Female	5.571429	13	2	522859.3	1822400	2430000	890502.77	860954.26
1668	26	Female	4.714286	17	2	390539.75	1242400	5158100	1108400	4860900
1671	31	Male	6	10	1	453254.55	1167600	2031400	568900.79	803300.55
1684	25	Female	6	22	6	289560.11	1132700	885601.07	690079.62	762685.15
1685	26	Female	6.5	25	1	228153.27	732207.3	1137700	254109.45	244262.79
1689	27	Female	6	17	1	46305.88	168001.6	97459.22	27212.89	43565.41
1690	25	Female	6.428571	6	2	742492.17	1733000	4487800	599928.75	856309.77
1697	22	Male	4.5	23	8	376776.86	819294.6	934890.83	425040.87	439483.51
1707	22	Female	5.952381	14	0	224916.79	838189	811574.7	259640.89	290230.11

ID	Age	Gender	CLH-S Score	Perceived Stress	ACE Score	OXY Saliva Level (pg/mL	a-MSH (pg/mL)	B Endorphin (pg/mL)	Neurotensin (pg/mL)	Substance P (pg/mL)
1711	30	Male	5.809524	15	4	14931.77	18221.69	17044.83	5330.64	3650.26
1723	24	Male	6.333333	17	0	13804.95	21477.81	18801.52	5328.71	3511.38
1728	26	Female	4.333333	15	0	2972.4	2045.6	3297.94	1111.74	621.69
1733	26	Female	4.428571	22	1	7235.43	4790.07	7936.77	2525.81	1474.67
1737	25	Female	5.571429	22	1	14550.87	21503.14	19039.09	5427.27	3504.55
1757	30	Female	6.380952	17	2	5440700	5938900	28550000	805479.8	539624.09
1766	28	Female	5.285714	13	4	29838.3	68618.35	46835.55	11192.4	11058.39
1780	27	Female	6	24	1	6071.06	4959.7	6919.68	1999.04	1212.04
1786	22	Female	4.571429	24	2	473460.8	1739600	4122600	699843.78	924807.92
1794	23	Male	4.714286	12	0	33830.82	84472.64	53881.57	14701.29	12667.74
1797	64	Female	5.5	27	3	1634.43	589.85	2031.32	592.36	338.36
1800	23	Female	4.380952	13	3	5364.83	7678.83	4766.63	1423.32	1021.34
1805	28	Female	5.952381	31	0	34122.66	111287.9	52500.96	17698.47	18246.42
1823	25	Male	5.238095	27	4	7232.37	5651.46	7380.75	2751.27	1433.37
1834	25	Male	5.142857	21	6	112103.44	586809.5	370666.42	246173.86	255106.41
1846	29	Male	5.952381	3	2	13217.2	17923.93	15408.13	5286.48	3644.85
1855	31	Female	4.952381	20	2	15070.75	18738.45	14799.76	5516.07	3263.85
1856	26	Female	6.5	13	6	8866.69	9340.09	10795.78	3004.49	2010.06
1863	33	Male	7	5	3	4219.85	2826.45	4497.32	1691.86	777.52
1864	25	Male	5.619048	8	1	24400.54	40417.44	30906.59	9028.69	7449.84
1873	26	Female	6	9	0	17043.91	24188.11	21104.61	6108.99	4954.36
1882	23	Female	7	19	0	3206.87	1746	4333.99	1453.29	736.52
1888	25	Male	4.952381	14	0	37653.85	106206.5	62917.86	17538.06	15657.94
1891	30	Male	5.571429	29	3	36227.3	80565.24	57504.63	14980.08	13834.15
1892	25	Female	5.904762	22	0	242668.06	727026.4	758737.86	193463.86	245770.49
1900	25	Female	5.619048	24	0	21244.08	46497.94	27998.81	9272.31	8110.04
1901	24	Male	4.666667	12	1	14078.33	20856.24	17399.15	5453.02	3923.51

ID	Age	Gender	CLH-S Score	Perceived Stress	ACE Score	OXY Saliva Level (pg/mL	a-MSH (pg/mL)	B Endorphin (pg/mL)	Neurotensin (pg/mL)	Substance P (pg/mL)
1905	25	Female	4.5	22	3	14256.59	16057.19	15942.5	4841.02	3492.62
1910	23	Female	6.047619	16	0	7390.53	32722.08	26038.32	7356.33	5757.21
1922	28	Female	5.52381	26	0	296702.52	7049.14	7734.7	2709.74	1634.9
1925	25	Female	6	26	3	68715.3	1416600	1412700	666642.64	693315.08
1933	33	Male	4.571429	21	7	24590.74	274469.3	141990.1	45004.55	61064.52
1938	61	Female	5.761905	4	0	2971.72	40727.19	33179.17	9190.05	7930.75
1944	26	Female	6.333333	17	1	120085.2	2095.79	3773.29	1077.51	682.02

Supplemental Table S4 provides the available demographic information for all participants and their corresponding survey scores as well as neuropeptide levels. The parameters included in this table summarize the factors that were taken into account in our data analysis.

Supplemental Figure S1 – Proposed Neuropeptide Molecular Mechanisms

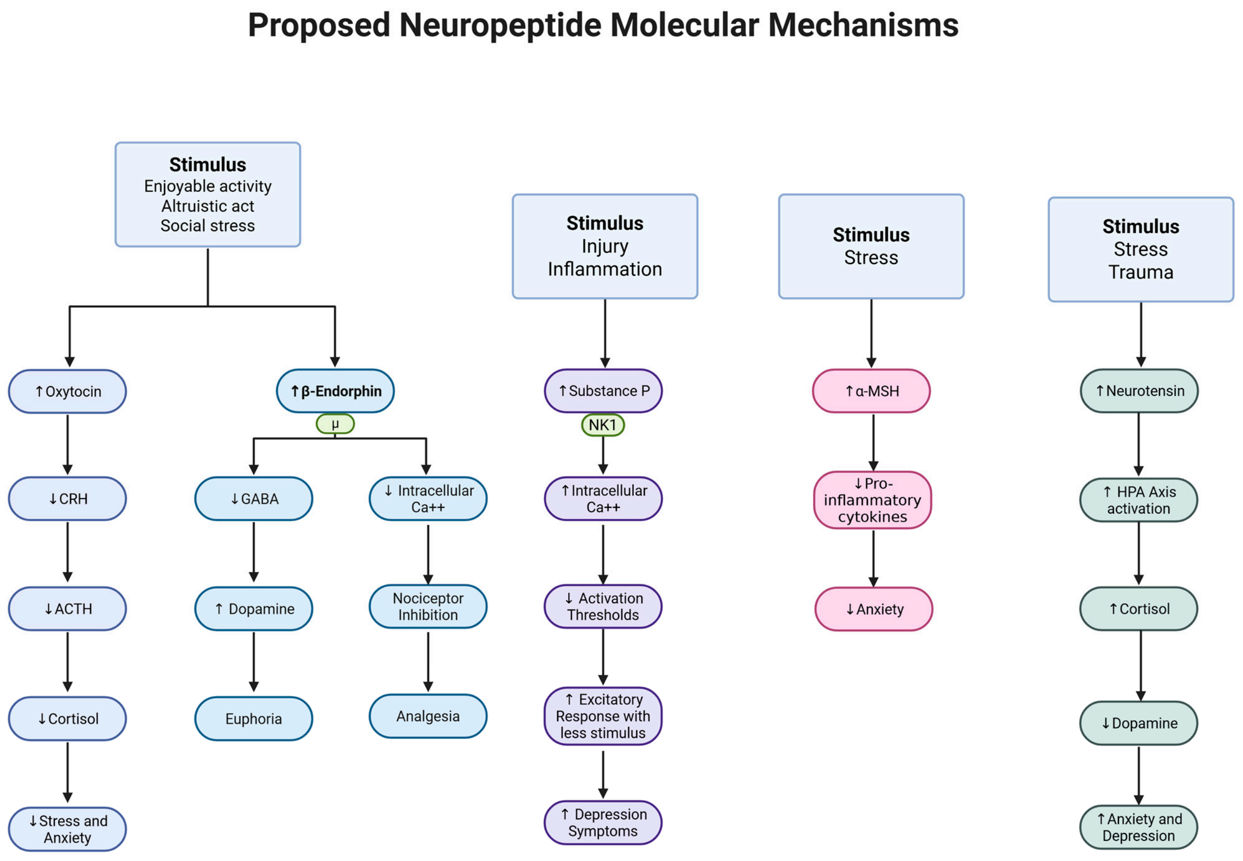

Supplemental Figure S1 illustrates the proposed molecular mechanisms for each neuropeptide and corresponding psychological condition.

Supplemental Table S5 – ELISA Data Sensitivity and Specificity

Analyte	Minimum Detectable Concentration (pg/mL)	Cross Reactivity
α-MSH	63	Negligible cross-reactivity between antibodies for an analyte and any of the other analytes
-Endorphin	85	Negligible cross-reactivity between antibodies for an analyte and any of the other analytes
Neurotensin	43	Negligible cross-reactivity between antibodies for an analyte and any of the other analytes
Oxytocin	63	Negligible cross-reactivity between antibodies for an analyte and any of the other analytes
Substance P	10	Negligible cross-reactivity between antibodies for an analyte and any of the other analytes

Supplemental Table S5 describes the sensitivity and specificity of the ELISA assay used for each neuropeptide.
